# Remote Ischemic Preconditioning Ameliorates Acute Kidney Injury due to Contrast Exposure in Rats through Augmented O-GlcNAcylation

**DOI:** 10.1155/2018/4895913

**Published:** 2018-08-13

**Authors:** Jiachang Hu, Yimei Wang, Shuan Zhao, Jing Chen, Shi Jin, Ping Jia, Xiaoqiang Ding

**Affiliations:** ^1^Department of Nephrology, Zhongshan Hospital, Fudan University, Shanghai 200032, China; ^2^Shanghai Medical Center of Kidney, Shanghai 200032, China; ^3^Shanghai Institute of Kidney and Dialysis, Shanghai 200032, China; ^4^Shanghai Key Laboratory of Kidney and Blood Purification, Shanghai 200032, China; ^5^Hemodialysis Quality Control Center of Shanghai, Shanghai 200032, China

## Abstract

Remote ischemic preconditioning (RIPC) is an adaptive response, manifesting when local short-term ischemic preconditioning reduces damage to adjacent or distant tissues or organs. O-linked *β*-N-acetylglucosamine (O-GlcNAc) glycosylation of intracellular proteins denotes a type of posttranslational modification that influences multiple cytoplasmic and nuclear protein functions. Growing evidence indicates that stress can induce an acute increase in O-GlcNAc levels, which can be cytoprotective. The current study aimed to determine whether RIPC can provide renoprotection against contrast-induced acute kidney injury (CI-AKI) by augmenting O-GlcNAc signaling. We established a stable model of CI-AKI using 5/6 nephrectomized rats exposed to dehydration followed by iohexol injection via the tail vein. We found that RIPC increased UDP-GlcNAc levels through the hexosamine biosynthetic pathway as well as global renal O-GlcNAcylation. RIPC-induced elevation of O-GlcNAc signaling ameliorated CI-AKI based on the presence of less tubular damage and apoptosis and the amount of reactive oxygen species. In addition, the use of alloxan, an O-GlcNAc transferase inhibitor, and azaserine, a glutamine fructose-6-phosphate amidotransferase inhibitor, neutralized the protective effect of RIPC against oxidative stress and tubular apoptosis. In conclusion, RIPC attenuates local oxidative stress and tubular apoptosis induced by contrast exposure by enhancing O-GlcNAc glycosylation levels; this can be a potentially useful approach for lowering the risk of CI-AKI.

## 1. Introduction

The number of patients at risk of developing contrast-induced acute kidney injury (CI-AKI) is growing, owing to the popularity of iodinated contrast media (CM) use for different imaging studies. Existing studies suggest that CI-AKI develops in 2%–50% of individuals undergoing coronary artery angiography, and the incidence varies depending on the presence of different risk factors, including advanced age, chronic kidney disease (CKD), and diabetes [1]; among those who receive contrast-enhanced computed tomography, the incidence can be higher than 10% [2]. Currently, a pharmacologic approach for preventing or treating CI-AKI has been unsuccessful, based on a recent randomized controlled trial showing that a no-hydration protocol for CI-AKI prophylaxis can be noninferior to and more cost-saving than prophylactic intravascular volume expansion with normal saline in high-risk patients.

Remote ischemic preconditioning (RIPC) is a revolutionary therapeutic modality, consisting of intermittent practices of repeated ischemia coupled with reperfusion, which was found to protect distant organs from developing such injury. This approach has been controversial since several recent randomized controlled trials reported contradictory findings [3, 4]. In a meta-analysis [5], we also proved that RIPC could not prevent ischemia/reperfusion-induced AKI (IR-AKI) after cardiac surgery, but it significantly reduced the CI-AKI incidence from 13.5% to 6.5%. RIPC is thought to activate several pathways, including systemic anti-inflammatory, neuronal, and humoral signaling [6], but the underlying mechanisms are likely complex and have not been fully elucidated.

Glycans are the most abundant and complex group of molecules in living organisms. Frequently attached to proteins to form simple and complex glycoconjugates; in a process defined N-linked *β*-N-acetylglucosamine (N-GlcNAc) and O-linked *β*-N-acetylglucosamine (O-GlcNAc), they regulate several aspects of protein function and participate in many key physiological processes [7]. O-GlcNAc transfer denotes a unique mechanism that regulates protein modifications and governs multiple protein functions through the dynamically regulated cycles constituted by the hexosamine biosynthetic pathway (HBP), O-GlcNAcase (OGA), and O-GlcNAc transferase (OGT). In studies involving O-GlcNAc, it is a common practice to alter UDP-GlcNAc levels by inhibiting HBP enzymes. This type of protein alteration reportedly occurs for a multitude of proteins and has been implicated in the pathogenesis of many diseases, such as cancer, diabetes, and neurodegenerative disorders. Growing evidence reveals that the stress-induced elevation in O-GlcNAc levels is cytoprotective in the short term. We [8] also showed previously that the upregulation of O-GlcNAc levels via glucosamine significantly reduces reactive oxygen species generation and attenuates apoptosis related to CI-AKI in rats. In fact, not only local ischemic preconditioning [9] but also RIPC [10] may even achieve organ protection through the O-GlcNAc glycosylation pathway, but the underlying mechanisms are still unclear.

In this study, we hypothesized that RIPC, as an intrinsic protective mechanism, exerts its renoprotective effect by stimulating O-GlcNAc signaling via the regulation of HBP and OGT activity. This upregulation of O-GlcNAc signaling following RIPC might ameliorate renal reactive oxygen species generation and lower tubular apoptotic severity in the renal parenchymal induced by CI-AKI.

## 2. Materials and Methods

### 2.1. Materials Involved in the Current Study

Azaserine (AZA) and alloxan monohydrate (AX) were obtained from Sigma-Aldrich Corp. (St. Louis, MO, USA); AZA and AX were subsequently diluted in saline. We used a nonionic CM (iohexol, low-osmolar; 350 mg I/mL, 844 mOsm/kg water as well as 10.4 cPs at 37°C GE Healthcare, Shanghai, China) in our experiments. We purchased a Western blot detection kit for detecting O-GlcNAc (monoclonal antibody; MAb CTD110.6; Thermo Fisher Scientific, Rockland, IL, USA) and obtained GAPDH antibody from Cell Signaling (Danvers, MA, USA). Antibodies against Bcl-2, BAX, and cleaved caspase-3 were purchased from Abcam (Cambridge, MA, USA).

### 2.2. Animals and Grouping

We purchased male Sprague-Dawley rats (~200 g) from the Animal Center of Fudan University, Shanghai, China. Animals were acclimatized for 1 week before undergoing experiments. We performed 5/6 nephrectomy (Nx) on anesthetized rats using intraperitoneal 4% sodium pentobarbital (40 mg/kg). We have used this procedure to establish a valid CI-AKI model by 6 weeks after the 5/6 Nx procedures, as described previously [8, 11, 12]. After dehydration for 48 h, 10 mL/kg (3.5 g I/kg) iohexol was injected via the tail vein. RIPC was also carried out according to the procedures described in our previous study [12]. After being anesthetized with intraperitoneal 4% sodium pentobarbital injection, rats received transverse incisions over the femoral triangle, with right lateral femoral arteries uncovered and clamped for four cycles of ischemia-reperfusion (5/5 min). After RIPC, the femoral wound was closed. The sham operation group underwent the same operation except for the clamping of the femoral artery.

The Animal Care and Use Committee of Fudan University approved the experimental protocols. Experiments in this study were done in adherence to the Guidelines for the Care and Use of Laboratory Animals, National Academy Press (NIH Publication No. 85-23, revised 1996).

#### 2.2.1. Confirmation of CI-AKI Model Findings

About 0.3 mL blood was obtained via the tail vein and processed for detecting serum creatinine (SCr) to observe the trends in renal function after the 5/6 Nx procedure at the following time points: before and 1, 2, 4, and 6 weeks after the operation. In total, 24 rats with similar body weight and SCr levels between 1.5 mg/dL and 2.5 mg/dL were selected 6 weeks after the 5/6 Nx procedure. They were divided randomly into the following groups (*n* = 6 in each group): (1) normal saline (NS) group, which received 10 mL/kg saline after 48 h of dehydration; and (2) CM group, which received 10 mL/kg iohexol after 48 h of dehydration. We assessed SCr and renal hematoxylin and eosin (H&E) staining at 24 and 72 h after the last injection.

#### 2.2.2. Renoprotective Effect of RIPC and Its Influence on O-GlcNAc Signaling

Twenty-four rats with conditions similar to those described above were divided randomly into the following four groups (*n* = 6): sham + NS, RIPC + NS, sham + CM, and RIPC + CM groups. RIPC or sham procedures were performed before iohexol or normal saline intravenous administration. We assessed SCr, urinary and serum neutrophil gelatinase-associated lipocalin (NGAL), terminal deoxynucleotidyl transferase-mediated dUTP nick-end labeling (TUNEL), H&E staining, and CellROX® Green-based ROS detection using renal tissues at 24 h after the last treatment dose. Renal tissues were cryopreserved at −80°C for use in subsequent studies.

#### 2.2.3. Inhibitor Effect of OGT on O-GlcNAc Expression Levels and the Renoprotective Effect of RIPC

The activity of OGT may be a potential target of RIPC [10]. Alloxan (AX), an OGT inhibitor, has been used to attenuate global protein O-GlcNAcylation levels [13]. Twenty-four rats with conditions similar to those described above were divided randomly into the following four groups (*n* = 6): (1) sham + CM group, which received sham procedures and vehicle with 10 mL/kg iohexol; (2) RIPC + CM group, which received RIPC and intraperitoneal 10 mL/kg iohexol; (3) RIPC + AX + CM group, receiving RIPC and intraperitoneal 50 mg/kg AX [8, 14], diluted in normal saline to a final concentration of 16.7 mg/mL, 0.3 mL/100 g body weight, and 10 mL/kg iohexol; and (4) sham + AX + CM group, receiving sham procedures and intraperitoneal 50 mg/kg AX and 10 mL/kg iohexol. Intraperitoneal vehicle and AX injection were done 5 minutes before intravenous iohexol administration. We assessed the SCr, urinary and serum NGAL, H&E staining, and TUNEL staining of renal tissues at 24 h after the last treatment dose. Renal tissues were cryopreserved in −80°C for the subsequent studies.

#### 2.2.4. GFAT Inhibitor Effect on O-GlcNAc Signaling and the Renoprotective Effect of RIPC

Both the OGT pathway and HBP may play a role in the protective effect related to RIPC. Glutamine fructose-6-phosphate amidotransferase (GFAT), in particular, is a crucial HBP enzyme that is rate-limiting. Thus, we used AZA as a GFAT inhibitor to evaluate the role of HBP in the effect of RIPC [15]. Twenty-four rats with conditions similar to those described above were divided randomly into the following four groups (*n* = 6): (1) sham + CM group, which received sham procedures and vehicle with 10 mL/kg iohexol; (2) RIPC + CM group, which received RIPC and intraperitoneal 10 mL/kg iohexol; (3) RIPC + AZA + CM group, which received RIPC and intraperitoneal 10 mg/kg AZA, diluted in normal saline resulting in a final concentration of 10 mg/mL, 0.1 mL/100 g body weight [15], and 10 mL/kg iohexol; and (4) sham + AZA + CM group, which received sham procedures and intraperitoneal 10 mg/kg AZA and 10 mL/kg iohexol. Intraperitoneal vehicle and AZA injection were done 5 min before intravenous iohexol administration. We assessed SCr, urinary and serum NGAL, H&E staining, and TUNEL staining of renal tissues at 24 h after the last treatment dose. Renal tissues were cryopreserved in −80°C for the subsequent studies.

### 2.3. Serum and Urinary Biomarker Quantification

We collected about 0.3 mL blood from the tail vein prior to the development of AKI and obtained 1 mL blood after puncturing the abdominal aorta during sacrifice 24 h after CM injection. We collected serum after clotting at 4°C overnight and centrifugation at 2000g for 15 min. SCr concentrations were tested using the QuantiChrom™ Creatinine Assay Kit and Urea Assay Kit (BioAssay Systems, Hayward, CA, USA). We used bladder puncture to collect urine 30 min after anesthesia 24 h after CM injection. We determined urinary and serum NGAL concentrations utilizing a rat NGAL enzyme-linked immunosorbent assay (ELISA) kit from Abcam (Cambridge, MA, USA).

### 2.4. H&E Staining

We immersed renal tissues in 10% neutral-buffered formalin for more than 24 h, followed by paraffin embedding. We used a microtome to prepare tissue sections of 3 *μ*m thickness and applied H&E staining for evaluation. Histological changes, mainly as detachment and foamy degeneration of tubular cells in the corticomedullary junction and outer medulla, were evaluated semiquantitatively according to a scoring system based on the percentage of damaged tubules per field [16]: very severe (>75%), 4; severe (<75%), 3; moderate (<50%), 2; mild (<25%), 1; and no injury, 0. Two pathologists, who were blinded to the study protocol, interpreted the pathologic findings under light microscopes (Leica DM 6000 B; Leica Microsystems, Wetzler, Germany). They randomly selected 10 high-magnification fields (×200) of the corticomedullary boundary zone to evaluate the tubular injury score.

### 2.5. TUNEL Assay

The TUNEL assay was conducted on paraffin sections of the renal corticomedullary boundary zone, using a commercial kit (In Situ Cell Death Detection Kit; KeyGEN Biotech, Nanjing, China), to evaluate the severity of tubular apoptosis. We determined the TUNEL-positive cell counts and total cell counts in renal sections under fluorescence microscopy (Olympus BX51, Japan). We assessed the TUNEL-positive cells at 400x magnification over 10 fields and described the results as percentages of total cells.

### 2.6. CellRox for Detecting Reactive Oxygen Species (ROS)

The detectable amount of renal tubular ROS can be a surrogate for the severity of oxidative stress during CI-AKI. We tested ROS levels on frozen renal sections using CellROX Green Reagent from Thermo Fisher Scientific (Rockland, IL, USA); the signal emitted from CellROX Green Reagent will localize to the nucleus and mitochondria upon oxidation. This reagent has been used to estimate ROS [8, 17, 18] produced by molecules such as angiotensin II, lipopolysaccharide, menadione, and nefazodone. We permeabilized frozen sections in 0.5% Triton® X-100 for 10 min and incubated slides with CellRox (5 *μ*M) for 30 min at 37°C. These slides were then washed in phosphate-buffered saline (PBS). DAPI (2 mg/mL) was used as the nuclear counterstain. After a PBS wash, we acquired images using fluorescence microscopes (Olympus BX51, Japan) and analyzed the results with ImageJ (V1.44, http://rsbweb.nih.gov/ij/) software, followed by image merging and synthesis of composite figures. The ROS-positive cells were counted at 400x magnification over 10 fields, with results described using percentages of total cells.

### 2.7. Renal Malondialdehyde (MDA) and Superoxide Dismutase (SOD) Activities

Renal cortical homogenate supernatants were tested for MDA and SOD levels using the following kits (MDA and SOD assay kits; KeyGEN Biotech, Nanjing, China). Protein concentrations were evaluated using a BCA protein assay kit (KeyGEN Biotech, Nanjing, China). MDA levels are expressed as nmol/mg protein, and tissue SOD activities are shown as U/mg protein.

### 2.8. Western Blotting

We homogenized renal tissues in ice-cold lysis buffer consisting of protease inhibitor cocktail (Beyotime Biotechnology, China). The supernatants were centrifuged at 12000g for 30 min at 4°C, yielding total protein extract, and we measured protein concentrations using a BCA protein assay kit (KeyGEN Biotech, Nanjing, China). Subsequently, we loaded 40~80 *μ*g total protein per lane onto a Tris/HCl SDS polyacrylamide gel with 10% gradient, performed electrophoresis, and transferred samples to another polyvinylidene difluoride membrane, followed by blocking with a solution containing 5% skim milk powder in 1X TBS for 1 h. The membrane was subsequently incubated with primary antibodies against CTD110.6 (mouse monoclonal 1 : 1000), Bcl-2 (mouse monoclonal 1 : 1000), BAX (mouse monoclonal 1 : 1000), cleaved caspase-3 (mouse monoclonal 1 : 500), and GAPDH (rabbit monoclonal 1 : 2000) at 4°C overnight, washed with TBS-T three times for 15 min, and reincubated with rabbit or goat secondary antibodies conjugated with horseradish peroxidase (1 : 5000 dilution; Kang Chen Biotechnology, Guangzhou, China) for 1 h at room temperature. The blot was washed three times (10 min each), and we used an ECL Western blotting detection reagents (Thermo Fisher Scientific, Pittsburgh, PA, USA) and a LAS-3000 detection system to detect antigen-antibody complexes. We also used ImageJ to analyze band intensities. The results were normalized to GAPDH levels.

### 2.9. Detection of UDP-GlcNAc

The concentration of UDP-GlcNAc in renal cortical homogenate supernatants was quantified using UDP-Glo™ glycosyltransferase assay (Promega, Madison, WI, USA). This is a homogeneous, single-reagent-addition method to rapidly detect UDP formation, including UDP-GlcNAc, in glycosyltransferase reactions. An equal volume of UDP detection reagent is added to convert the UDP-GlcNAc to ATP and generate light in a luciferase reaction. Luminescence was recorded using the Infinite® M1000 PRO plate-reader luminometer (Tecan, Switzerland), and UDP-GlcNAc concentration was calculated using a standard curve technique.

### 2.10. Statistical Analysis

GraphPad Prism 5.03 (GraphPad Software, San Diego, CA) was used to perform and display the statistics. Continuous variables were expressed as mean ± standard deviation, and the statistical significance was estimated by t-test between two groups or one-way analysis of variance (ANOVA) accompanied by Tukey's multiple comparisons for parametric tests. However, the tubular injury scores were compared using Kruskal-Wallis test accompanied by Dunn's multiple comparisons for the nonnormal distribution. Statistically significant differences were defined as a *P* value < 0.05.

## 3. Results

### 3.1. Successful Establishment of the Rat CI-AKI Model

**Figure 1:**
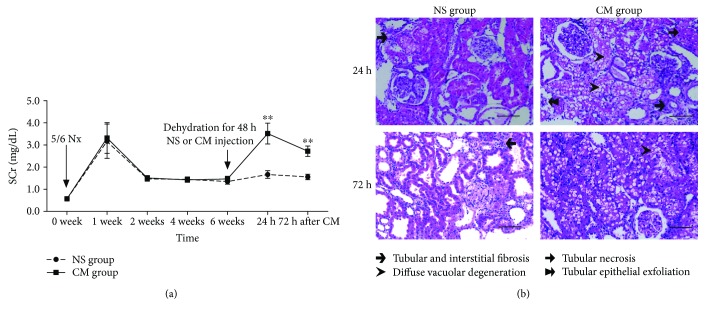
Establishing a CI-AKI rat model based on 5/6 Nx. (a) The dynamic change in SCr before and 2, 4, and 6 weeks after the 5/6 Nx procedure and the increased SCr after injection of 10 mL/kg iohexol. (b) Representative photomicrographs of tubular injury at 24 and 72 h after CM injection. Scale bars represent 100 *μ*m (original magnification, ×200). ^∗∗^*P* < 0.01 compared with the NS group.

A reliable animal model of CI-AKI is most important for bridging the “bench-to-bedside” gap. As shown in Figure 1, we successfully established a rat CI-AKI model based on 5/6 Nx, iohexol injection and dehydration; the dynamic SCr changes before and 2, 4, and 6 weeks after the 5/6 Nx procedures are shown in Figure 1(a). At 6 weeks, the rats received a 10 mL/kg iohexol injection after 48 h of dehydration. Compared to the NS group, the CM group exhibited significantly increased SCr levels 24 h after CM injection. Although there was a downward trend in SCr at 72 h after CM injection, the SCr level did not yet approach the primary level. The renal pathology in the NS group only showed a reduction in renal glomerular, tubular, and interstitial fibrosis (Figure 1(b)). In addition to these pathological manifestations of chronic kidney disease (CKD), the CI-AKI model also presented tubular injury induced by CM, manifesting as diffuse vacuolar degeneration and tubular necrosis, accompanied by tubular epithelial exfoliation.

### 3.2. RIPC Ameliorates Oxidative Stress, Apoptosis, and Renal Injury in CI-AKI

**Figure 2:**
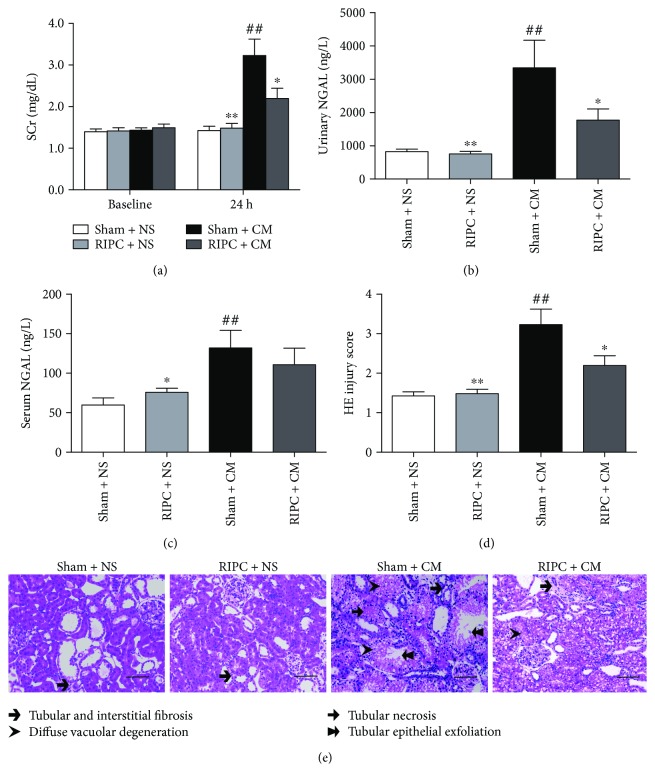
Renoprotection against CI-AKI by RIPC in rats. RIPC significantly lowered SCr (a) and urinary NGAL (b) levels at 24 h after CM injection; RIPC also decreased the level of serum NGAL but without a significant difference (c). (d, e) Representative photomicrographs of kidney tissue sections and the injury scores. Scale bars represent 100 *μ*m (original magnification, ×200). ^##^*P* < 0.01 compared with the sham + NS group; ^∗^*P* < 0.05, ^∗∗^*P* < 0.01 compared with the sham + CM group; *n* = 6.

**Figure 3:**
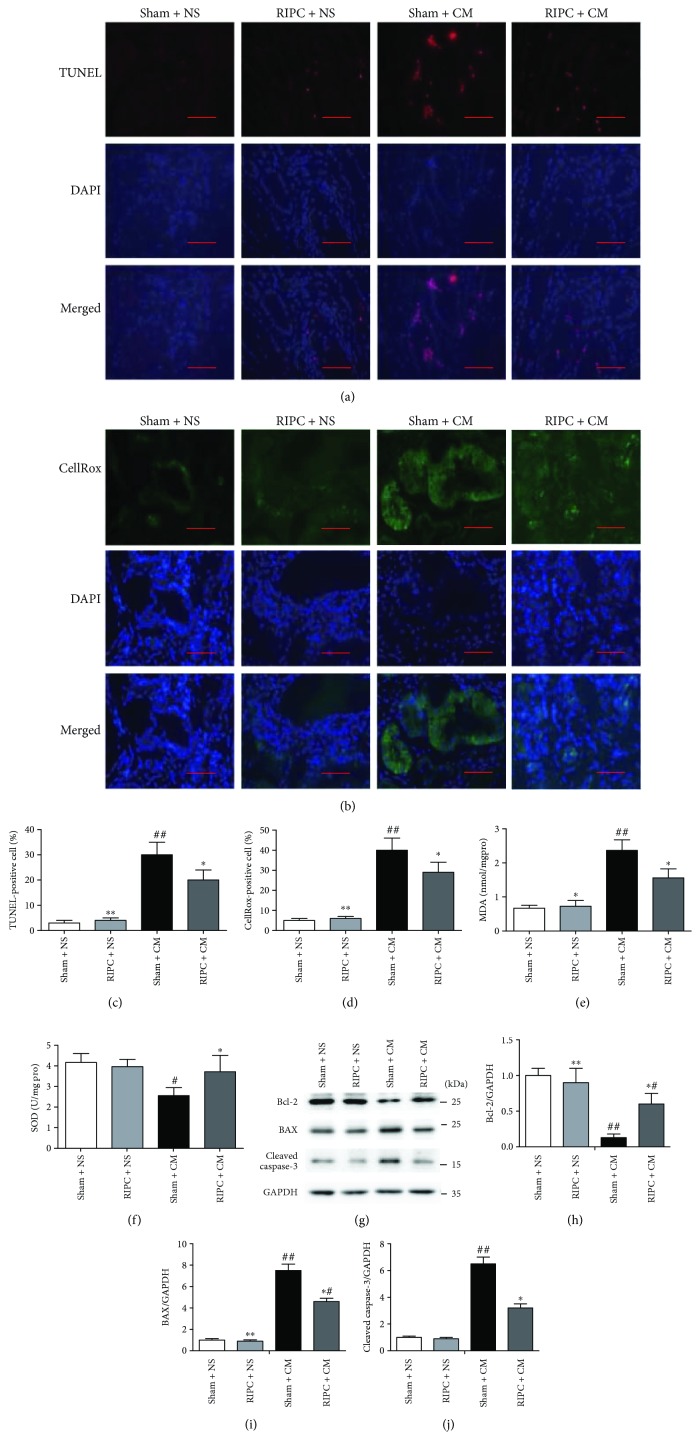
RIPC lowers the levels of apoptosis and oxidative stress in the rat CI-AKI model. (a, c) Quantitative analysis of TUNEL-positive cells and the characteristic photomicrographs of renal tissues using immunofluorescent labeling (red) for TUNEL. DAPI was used to counter-stain the nuclei (blue), with TUNEL-positive staining in nuclei. Scale bars represent 50 *μ*m (original magnification, ×400). (b, d) Quantitative analysis of CellRox-positive cells and the characteristic photomicrographs of renal tissues using immunofluorescent labeling (green) for ROS. Blue, DAPI; nuclear and mitochondrial CellRox-positive staining; scale bars represent 50 *μ*m (original magnification, ×400). Renal MDA (e) and SOD (f) concentrations as well as protein concentrations utilized as the comparison standard. The MDA level is expressed as nmol/mg protein, and SOD activities of renal tissues are expressed as U/mg protein. (g–j) Representative immunoblots and statistical analysis of Bcl-2 (H), Bax (i), and cleaved caspase-3 (j) in renal lysates. ^#^*P* < 0.05, ^##^*P* < 0.01 compared with the sham + NS group; ^∗^*P* < 0.05, ^∗∗^*P* < 0.01 compared with the sham + CM group; *n* = 6.

As an endogenous protective mechanism, RIPC ameliorated renal damage induced by iohexol exposure, manifesting as lower SCr and urinary NGAL (*P* < 0.05, Figures 2(a) and 2(b)) levels and lower HE injury scores (*P* < 0.05, Figures 2(d) and 2(e)) than those in the sham + CM group. RIPC nonsignificantly decreased the serum NGAL levels. TUNEL staining revealed that the RIPC + CM group had a significantly lower percentage of apoptotic cells than the sham + CM group (*P* < 0.05) (Figures 3(a) and 3(c)). RIPC significantly increased the level of Bcl-2, decreased the levels of BAX, and cleaved caspase-3 (Figures 3(g)–3(j)), also indicating its antiapoptosis effect. ROS assays using CellROX oxidative stress reagents showed that less ROS accumulation occurred in the RIPC + CM group than in the sham + CM group (*P* < 0.05, Figures 3(b) and 3(d)). Moreover, significantly less oxidative stress, presenting as lower MDA (*P* < 0.05, Figure 3(e)) and higher SOD (*P* < 0.05, Figure 3(f)) levels, was observed in the RIPC + CM group compared with the sham + CM group.

### 3.3. RIPC Increases Total Protein O-GlcNAcylation Levels in the Kidney

**Figure 4:**
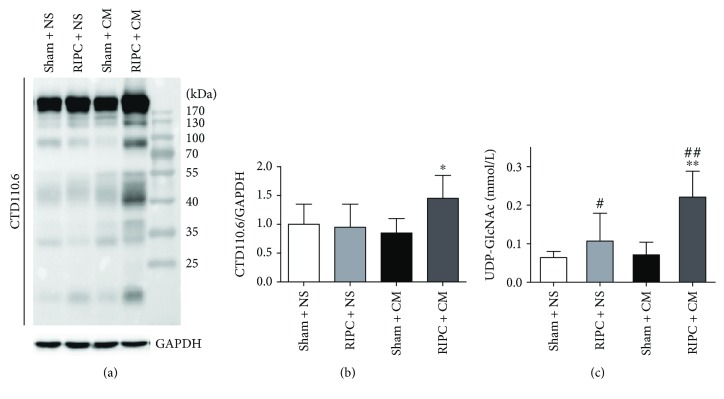
Effect of RIPC on renal O-GlcNAc signaling. The immunoblots (a) and the quantitation (b) of O-GlcNAcylation levels of total protein in renal tissues at 24 h after iohexol injection. (c) RIPC significantly increased UDP-GlcNAc levels in renal cortical homogenate supernatants. ^#^*P* < 0.05, ^##^*P* < 0.01 compared with the sham + NS group; ^∗^*P* < 0.05, ^∗∗^*P* < 0.01 compared with the sham + CM group; *n* = 6.

RIPC significantly enhanced protein O-GlcNAcylation (*P* < 0.05) compared with that in the sham + CM group (Figures 4(a) and 4(b)), although the total O-GlcNAcylated protein levels in the kidneys did not differ significantly between the sham + CM group and the sham + NS group. O-GlcNAcylation is strictly dependent on UDP-GlcNAc, which is both the substrate of OGT and the end-product of HBP. We found that RIPC increased the UDP-GlcNAc levels based on a comparison between the sham + NS group and the RIPC + NS group (*P* < 0.05) and also between the sham + CM group and the RIPC + CM group (*P* < 0.01), and the RIPC + CM group exhibited the highest UDP-GlcNAc levels (Figure 4(c)); this indicates that RIPC increased the levels of UDP-GlcNAc, especially under acute stress or injury.

### 3.4. Alloxan Decreases Global O-GlcNAcylation and Antagonizes Renoprotection of RIPC in CI-AKI

**Figure 5:**
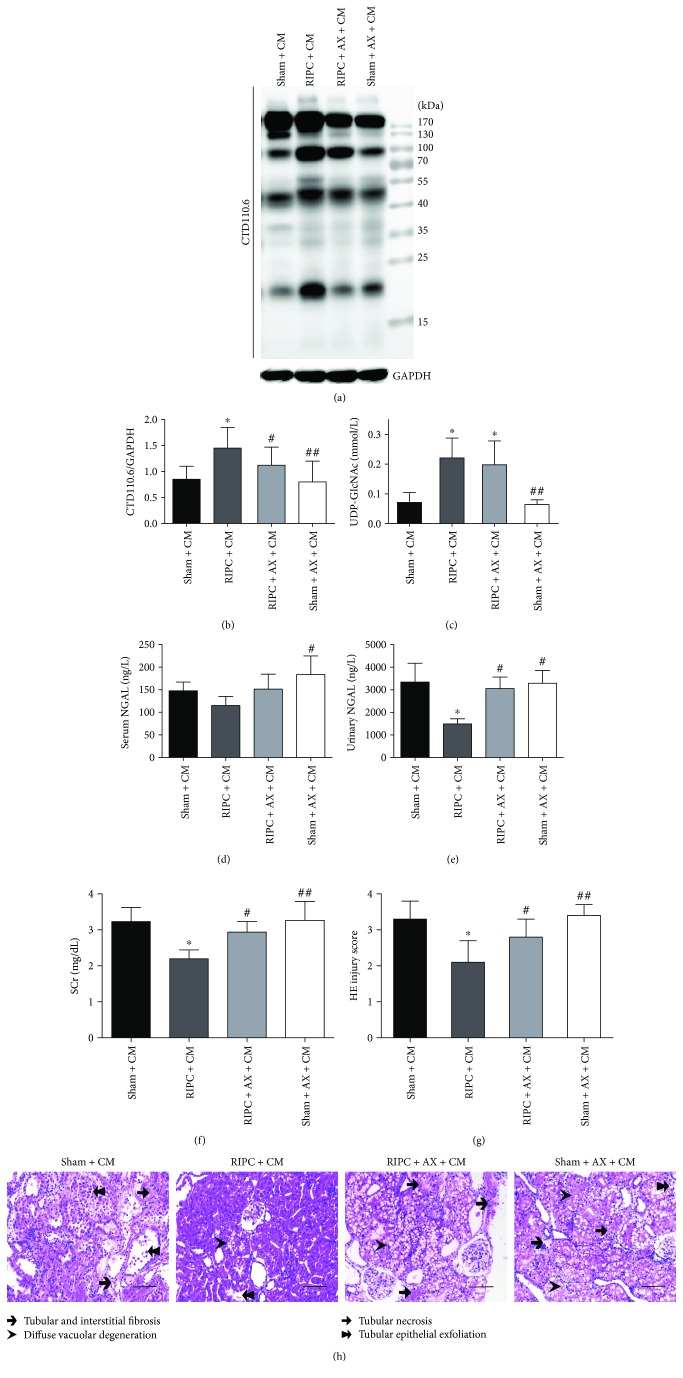
Alloxan decreases protein O-GlcNAcylation and blunts the renoprotection of RIPC in CI-AKI. (a, b) The immunoblots and the quantitation of the global renal O-GlcNAcylation levels from rats 24 h after iohexol treatment, (c) detecting UDP-GlcNAc concentrations in renal tissues and analyzing serum and urine biomarkers. Serum (d) and urinary (e) NGAL; SCr (f). (g, h) Photos of renal tubular injury. Scale bars represent 100 *μ*m (original magnification, ×200). ^#^*P* < 0.05, ^##^*P* < 0.01 compared with the RIPC + CM group; ^∗^*P* < 0.05 compared with the sham + CM group; *n* = 6.

To examine whether upregulated O-GlcNAc signaling plays a role in the renoprotection of RIPC against CI-AKI, AX was used as an OGT inhibitor. AX led to lower global protein O-GlcNAcylation in the RIPC + AX + CM (*P* < 0.05) and the sham + Ax + CM (*P* < 0.01) group compared with the RIPC + CM group (Figures 5(a) and 5(b)). However, AX had no effect on the increase in UDP-GlcNAc induced by RIPC (Figure 5(c)). Animals in the RIPC + AX + CM group showed marked renal function worsening (higher SCr and urinary NGAL, Figures 5(e) and 5(f)) and more severe renal morphologic damage (Figures 5(g) and 5(h)).

### 3.5. Azaserine Decreases the Level of UDP-GlcNAc and Global O-GlcNAcylation and Blunts the Renoprotection of RIPC in CI-AKI

**Figure 6:**
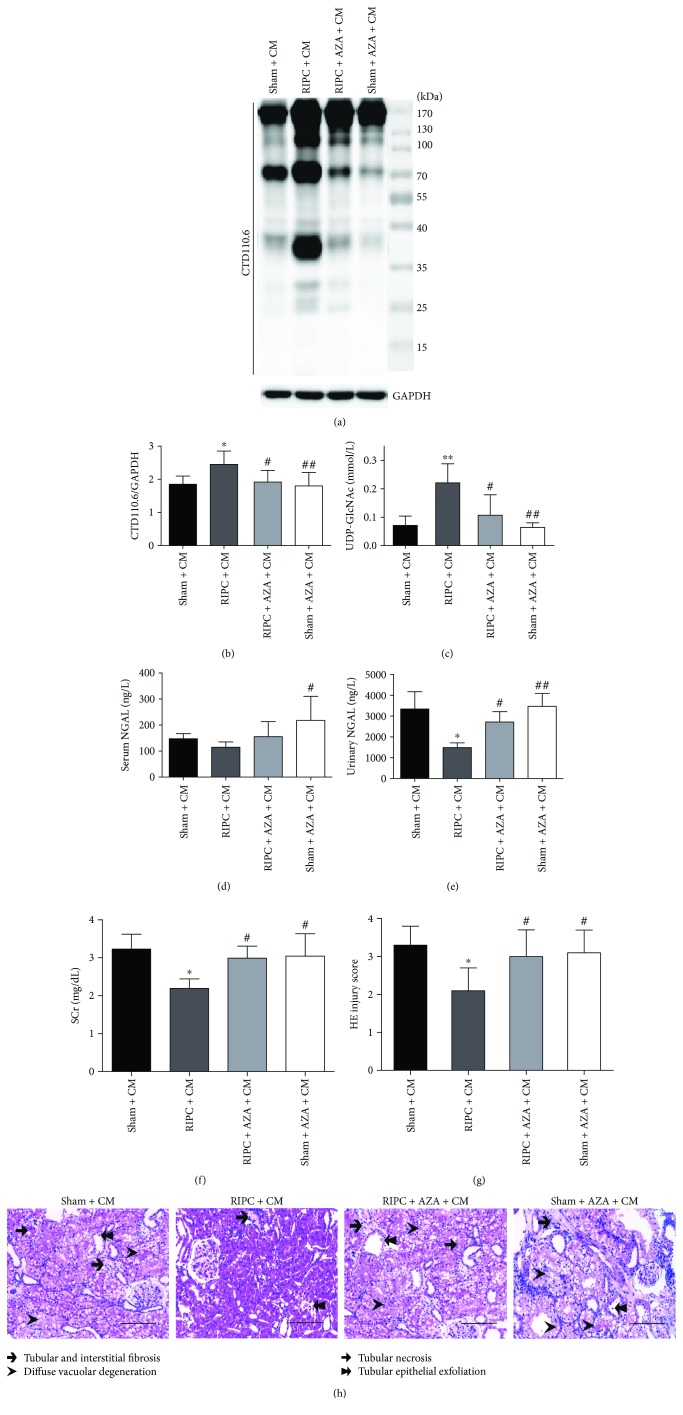
Azaserine decreases protein O-GlcNAcylation and blunts the renoprotection of RIPC in CI-AKI. (a, b) Immunoblots and quantitation of the global renal O-GlcNAcylation levels from rats 24 h after iohexol treatment. (c) Detection of UDP-GlcNAc concentrations in renal tissues and analysis of serum and urine biomarkers. Serum (d) and urinary (e) NGAL; SCr (f). (g, h) Photos of renal tubular injury. Scale bars represent 100 *μ*m (original magnification, ×200). ^#^*P* < 0.05, ^##^*P* < 0.01 compared with the RIPC + CM group; ^∗^*P* < 0.05, ^∗∗^*P* < 0.01 compared with the sham + CM group; *n* = 6.

To examine whether the upregulation of UDP-GlcNAc in response to RIPC plays a role in the renoprotection against CI-AKI, AZA was used as a GFAT inhibitor. We found that AZA decreased global protein O-GlcNAcylation levels in the RIPC + AZA + CM (*P* < 0.05) and the Sham + AZA + CM (*P* < 0.01) group compared with that in the RIPC + CM group (Figures 6(a) and 6(b)) and attenuated the upregulated UDP-GlcNAc levels induced by RIPC (*P* < 0.05, Figure 6(c)). Animals in the RIPC + AZA + CM group exhibited significantly worse renal function (higher SCr and urinary NGAL, Figures 6(e) and 6(f)) and more severe renal morphologic damage (Figures 6(g) and 6(h)).

### 3.6. Decreased O-GlcNAcylation Aggravates Oxidative Stress and Apoptosis

**Figure 7:**
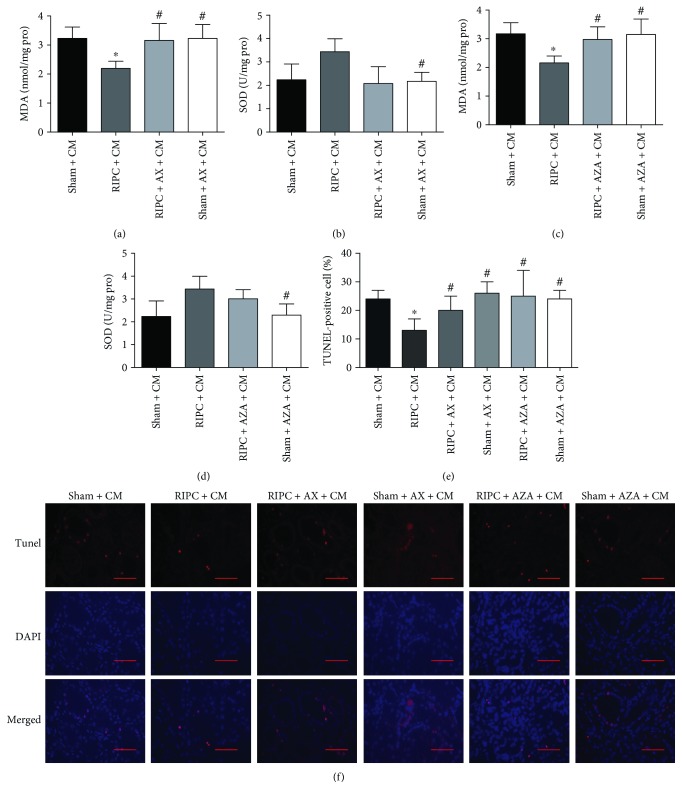
Both alloxan and azaserine blunted the antioxidative and antiapoptosis effects of RIPC in CI-AKI. Renal MDA (a, c) and SOD (b, d) concentrations were quantified, with protein concentrations utilized as the comparison standard. MDA levels, nmol/mg protein; tissue SOD activities, U/mg protein. (e, f) Quantitative analysis of TUNEL-positive cells and the characteristic photomicrographs of renal tissues using immunofluorescent labeling (red) of TUNEL. DAPI was used to counter-stain nuclei (blue), with TUNEL-positive staining in nuclei. Scale bars represent 50 *μ*m (original magnification, ×400). ^#^*P* < 0.05 compared with the RIPC + CM group; ^∗^*P* < 0.05 compared with the sham + CM group; *n* = 6.

Along with reducing O-GlcNAcylation, AX and AZA also blunted the antioxidative and antiapoptosis effects of RIPC. These two inhibitors increased the MDA level in the RIPC + AX and AZA + CM groups to levels higher than that in the RIPC + CM group (*P* < 0.05, Figures 7(a) and 7(c)), but had no significant influence on the SOD level (*P* > 0.05, Figures 7(b) and 7(d)). The percentages of TUNEL-positive cells (*P* < 0.05, Figures 7(e) and 7(f)) were also higher in the AX/AZA groups than in the RIPC + CM group.

## 4. Discussion

**Figure 8:**
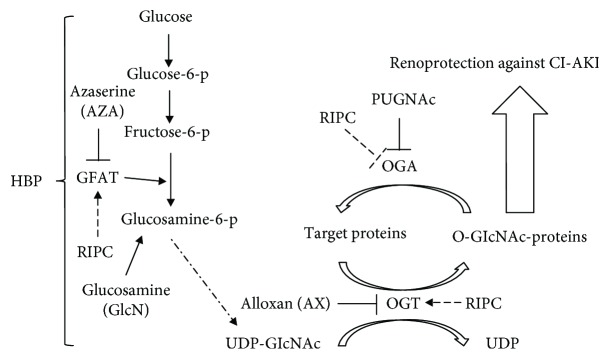
Schematic diagram of experimental hypothesis. RIPC increases the production of UDP-GlcNAc through the HBP, elevates the activity of OGT or inhibits the activity of OGA, increases the global O-GlcNAcylation in the kidneys, and ultimately exerts renoprotective effects under acute stress and injury.

In this study, we discovered that RIPC increased the production of UDP-GlcNAc through the HBP pathway and the global O-GlcNAcylation intensity in the kidneys. The upregulation of O-GlcNAc signaling induced by RIPC prevented renal injury caused by contrast media exposure, manifesting as attenuated tubular damages, less apoptosis, and lower oxidative stress. Further research revealed that AX and AZA blocked the antioxidative stress and antiapoptotic effects of RIPC and its renal protective effect. These results suggest that RIPC relieves renal oxidative stress and apoptosis induced by contrast media exposure by enhancing O-GlcNAc glycosylation levels, thereby serving as a promising strategy for lowering the risk of CI-AKI. A schematic diagram of the experimental hypothesis is shown in Figure 8.

We have previously proved that glucosamine relieves renal oxidative stress and apoptosis induced by contrast agent by enhancing the level of O-GlcNAc glycosylation and the activation of PI3K/Akt signaling pathways [8]. In this study, the upregulated O-GlcNAc signaling induced by RIPC exhibited renoprotection by reducing oxidative stress and apoptosis. Increased O-GlcNAc signaling may exert its protective role on multiple organs via various mechanisms including the prevention of mitochondrial permeability transition pore formation [19], the upregulation of heat shock protein 40 (HSP40) and HSP70 protein levels [20, 21], the activation of endoplasmic reticulum- (ER-) induced C/EBP homologous protein to prevent myocyte death [22], the phosphorylation of p38 mitogen-activated protein kinase [23], mitochondrial Bcl-2 [24], and the attenuation of nuclear factor-*κ*B (NF-*κ*B) signaling [25].

About 5% of glucose that enters the cells will be metabolized through the HBP [26], contributing to the production of UDP-GlcNAc. This is mainly controlled by the rate-limiting enzyme GFAT. GlcNAc can be transferred from UDP-GlcNAc to specific proteins, which is regulated by the action of two enzymes, OGT and OGA [27]. Various approaches have been utilized to increase O-GlcNAcylation and lower the risk of adverse outcomes. For example, the administration of glucosamine has been shown to improve trauma-related hemorrhage through the O-GlcNAc-dependent pathway in a rat model [25]. Similarly, intravenous administration of PUGNAc (O-(2-acetamido-2-deoxy-d-glucopyranosylidene) amino-N-phenyl carbamate), an OGA inhibitor, is capable of improving cardiac function and organ perfusion after trauma-related hemorrhage [28]. Overexpression of OGT via adenovirus infection was also reported to increase O-GlcNAc levels and attenuate posthypoxic damage to cardiomyocytes [23]. Finally, the administration of AZA not only decreased the rise in the O-GlcNAc level but also antagonized its cardioprotective effect [29, 30].

An isolated myocardial experiment [10] indicated that O-GlcNAc signaling participates in the mediation of RIPC-induced cardioprotection, and RIPC increases the activity of OGT but decreases the activity of OGA. Although the level of UDP-GlcNAc was not detected, AZA blocked the increase in O-GlcNAc, indicating that HBP may be involved in the augmented O-GlcNAc signaling induced by RIPC. In this study, we discovered that RIPC increased UDP-GlcNAc levels and raised the total O-GlcNAcylation in renal tissues, indicating that not only the activity of OGT but also the contribution of HBP participate in the regulation of O-GlcNAc signaling induced by RIPC. However, the intrinsic mechanisms are still unknown. A recent study [9] showed that spliced X-box binding protein 1 (Xbp1s), the most conserved component of the unfolded protein response (UPR), connected the UPR to the HBP for the purpose of protecting stress-sustaining cells. Xbp1s is also a direct transcriptional activator of the HBP. Ischemic preconditioning has been proven to activate the UPR by ER stress [31]. Another study [32] revealed that both RIPC and direct ischemic preconditioning upregulated antioxidant enzyme activity and ER stress-related proteins in rat skeletal muscles. Therefore, we speculated that RIPC might increase the levels of UDP-GlcNAc and O-GlcNAcylation through the regulation of the HBP by ER stress.

There are limitations within this study that need our attention. First, AX and AZA also lead to a significant number of off-target effects and generate cytotoxicity due to its ROS-inducing ability [33], but other OGT or GFAT inhibitors for use in rats are still unavailable. Second, the biological effects of upregulated O-GlcNAc levels are broad and complex, and the O-GlcNAcylated proteins should be identified using mass spectrometry or other methods, necessitating substantial research in the future. Third and most importantly, the intrinsic mechanisms linking RIPC and O-GlcNAc signaling were not fully elucidated. Nevertheless, further research may reveal the exact mechanisms of renoprotection of RIPC through O-GlcNAcylation and will provide a new theoretical basis of RIPC and therapeutic targets for the prevention and treatment of CI-AKI.

## 5. Conclusion

In summary, RIPC relieves renal oxidative stress and apoptosis related to contrast media exposure by enhancing the level of O-GlcNAc glycosylation, thereby protecting the kidneys as a promising strategy for preventing the occurrence of CI-AKI.

## Data Availability

The data used to support the findings of this study are available from the corresponding author upon request.

## Abbreviations

CI-AKI: Contrast-induced acute kidney injury
CM: Contrast media
RIPC: Remote ischemic preconditioning
O-GlcNAc: O-linked *β*-N-acetylglucosamine
HBP: Hexosamine biosynthetic pathway
OGA: O-GlcNAcase
OGT: O-GlcNAc transferase
GFAT: Glutamine fructose-6-phosphate amidotransferase
UPR: Unfolded protein response
AZA: Azaserine
AX: Alloxan monohydrate
NGAL: Neutrophil gelatinase-associated lipocalin
TUNEL: Terminal deoxynucleotidyl transferase-mediated dUTP nick-end labeling
ELISA: Enzyme-linked immunosorbent assay.
